# Combining superselective catheterization and electrochemotherapy: A new technological approach to the treatment of high-flow head and neck vascular malformations

**DOI:** 10.3389/fonc.2022.1025270

**Published:** 2022-11-29

**Authors:** Aljosa Krt, Maja Cemazar, Dimitrij Lovric, Gregor Sersa, Crt Jamsek, Ales Groselj

**Affiliations:** ^1^ Department of Otorhinolaryngology, Izola General Hospital, Izola, Slovenia; ^2^ Department of Experimental Oncology, Institute of Oncology Ljubljana, Ljubljana, Slovenia; ^3^ Faculty of Health Sciences, University of Primorska, Izola, Slovenia; ^4^ Department of Radiology, University Medical Centre Ljubljana, Ljubljana, Slovenia; ^5^ Faculty of Health Sciences, University of Ljubljana, Ljubljana, Slovenia; ^6^ Department of Otorhinolaryngology and Cervicofacial Surgery, University Medical Centre Ljubljana, Ljubljana, Slovenia; ^7^ Faculty of Medicine, University of Ljubljana, Ljubljana, Slovenia

**Keywords:** superselective catheterization, electrochemotherapy, high-flow malformations, AVM, bleomycin

## Abstract

**Introduction:**

The study aims to demonstrate a combination of superselective catheterization and electrochemotherapy as a feasible and effective new technological approach in treating high-flow vascular malformations of the head and neck region.

**Patients and methods:**

In the patient with high-flow arteriovenous malformation of the lower lip, superselective catheterization was performed under general anesthesia. The microcatheter was used to administer 750 IU BLM intra-arterially into the feeding vessel. The whole malformation surface was then covered with 15 applications of electric pulses using the plate electrode.

**Results:**

Excellent response, without functional or aesthetic deficits, was obtained in 10 weeks. During this period, debridement and necrectomy were performed regularly on follow-up visits. The pain was managed with oral paracetamol and sodium metamizole.

**Conclusion:**

Combining electrochemotherapy using bleomycin with superselective catheterization and arteriography is a feasible treatment option for high-flow vascular malformations in the head and neck region and could play a significant role in managing these challenging lesions.

## Introduction

Vascular malformations represent a heterogeneous group of vessel disorders, of which arteriovenous malformations (AVMs) are the rarest and the most resistant to treatment, despite different available treatment techniques (1–4). Currently, embolization and/or surgical excision are standard treatment options for AVM, with different outcome success rates (5). Electrochemotherapy (ECT) has recently emerged as an innovative local treatment for different malignant tumors (6, 7). ECT is an electroporation-based treatment that uses electric pulses to increase cytotoxic drug delivery into tumor cells. The most common cytotoxic drug administered in ECT is bleomycin (BLM), which is also used as a sclerosing agent in treating vascular malformations (6, 8–10). During ECT, BLM can be injected either intravenously or intratumorally. Electrochemotherapy of vascular malformations is also called electrosclerotherapy (10, 11).

In addition to the direct cytotoxic effect, the mode of action of ECT is also mediated by its impact on tumoral blood vessels. Electroporation, i.e., application of electric pulses provokes immediate but short-lasting vasoconstriction (vascular lock effect), which entraps BLM in the tumor vessels. Later, a more pronounced vascular disrupting effect occurs due to the cytotoxic effect of BLM on endothelial cells (6, 8, 12, 13).

There are several reports on good treatment outcomes of different vascular malformations: venous and arterial malformations, high- and low-flow, treatment-resistant venous malformations and capillary malformations (1, 10, 11, 14–16). BLM is given either directly into the malformation or intravenously. It is important to have the drug present in whole malformation at the time of electric pulse application (10, 11). The development of better coaxial microcatheters, guide wires and digital angiographic equipment have enabled more peripheral superselective catheterization of distal vessels and consequently allowed more selective vascular intervention. Such procedures are commonly used in the head and neck region, specifically for preoperative endovascular embolization of paragangliomas (17). However, combining the advantages of superselective catheterization and ECT might contribute to better outcomes in treating vascular lesions.

Our study aimed to combine superselective catheterization and ECT as a new technological approach in the treatment of high-flow vascular malformations. The feasibility and effectiveness of this concept were demonstrated in the case of a patient with one AVM localized on the lower lip.

## Patients and methods

### Patient characteristics

A 64-year-old female patient with a 10-year history of slow-growing red mass on the right side of the lower lip was hospitalized in the Department of Otorhinolaryngology and Cervicofacial Surgery, University Medical Centre Ljubljana, Slovenia, because of severe bleeding from the mass. In addition to regular therapy due to other comorbidities (diabetes mellitus type II, hypertension), the patient had regularly taken warfarin because of previous mitral and aortic valve replacement. Bleeding was managed with compression, and warfarin was replaced with low-molecular weight heparin to prevent thromboembolic events.

### Methods

**Figure 1:**
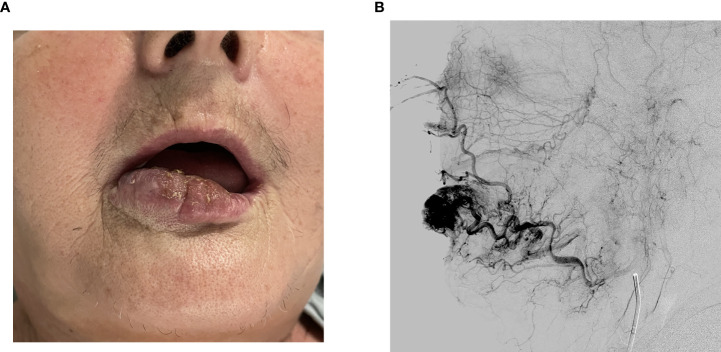
**(A)** Tumor on the right side of the lower lip before treatment. **(B)** Angiography showing a large and tortuous labial artery feeding the nidus.

**Figure 2:**
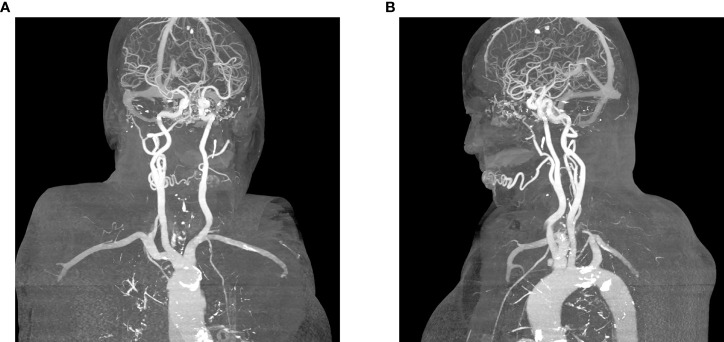
**(A)** Feeding artery and a single vein exiting the nidus, posterior projection, **(B)** Single vein exiting the nidus, lateral (left) projection.

A clinical exam revealed a 40 x 30 mm elastic malformation with palpable pulsation and central ulceration on the right side of the lower lip. A computed tomography angiography (CTA) scan demonstrated a 42 x 15 x 15 mm oval-shaped vascularized formation on the right side of the lower lip with a large and tortuous labial artery, feeding the malformation. The labial and facial artery diameters were 2.5 and 3.0 mm, respectively. A single vein exiting the nidus was seen. According to clinical and radiological characteristics, the vascular malformation was diagnosed as an AVM (**Figures 1**, **2**).

The patient was offered ECT combined with superselective catheterization as the potentially curative treatment option, and after detailed information about its advantages and drawbacks, she signed informed consent for treatment and publication of the data. This approach was proposed as treatment option based on reports of successful vascular malformation treatments with electrosclerotherapy (11, 16, 18). The National Ethics Committee approved the study (approval numbers 0120-135/2021/3 and 102/09/14).

## Results

**Figure 3:**
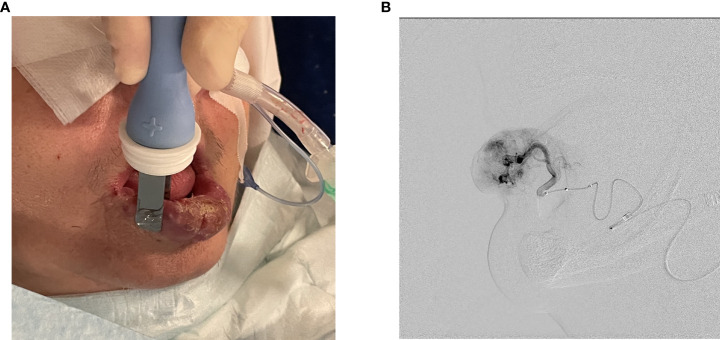
**(A)** Electric pulse administration with plate electrodes. **(B)** Angiography after ECT completion and balloon deflation demonstrating electroporation related vasoconstriction.

Treatment was performed under general anesthesia with nasotracheal intubation. After obtaining right common femoral artery access *via* a 6 Fr vascular sheath, right external carotid artery angiography was performed using a 5 Fr Infinity catheter (Cordis Europa N. V, Roden, The Netherlands). The procedure continued with selective catheterization and angiography of the right facial artery to adequately visualize AVM vascularization. Based on angiographic findings, superselective catheterization of the right labial artery was performed using a 2.6 Fr Headway27 microcatheter (MicroVention Deutschland GmbH, Düsseldorf, Germany). A Scepter C occlusion balloon (MicroVention Deutschland GmbH, Düsseldorf, Germany) was inflated 2 cm proximal to AVM in the inferior labial artery to achieve low-flow conditions. At that point, a microcatheter was used to administer 750 IU BLM directly into the feeding vessel. Thereafter, without delay, the whole malformation’s surface was covered with 15 applications of electric pulses using plate electrodes with an 8 mm gap between them. Each application consisted of 8 electric pulses with an amplitude of 1300 V/cm and a duration of 100 μs at a repetition frequency of 5 kHz delivered by the electric pulse generator Cliniporator Vitae^®^ (IGEA, Carpi, Italy). After ECT completion, balloon was deflated and electroporation related vasoconstriction was observed on angiography (**Figure 3**).

**Figure 4:**
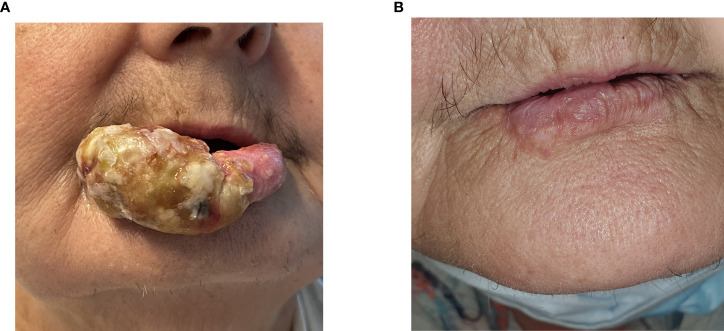
**(A)** 3 weeks post-treatment. **(B)** 18 months post-treatment.

The delivery of electric pulses was successful, and no heart rhythm disturbances or abnormalities were detected during the procedure. The next day, significant swelling of the treated area was observed, and suppuration developed in the following three days. The patient reported moderate pain that lasted one week after the treatment and was well controlled by oral paracetamol and sodium metamizole. The patient was discharged seven days after the procedure. Hospitalization was proposed solely for study and observation reasons and would otherwise not be necessary. During the regular follow-up visits, swelling and suppuration of the treated area were observed. Both events were grade III according to the Common Terminology Criteria for Adverse Event version 5 (CTCAEv5) (19). Debridement was performed over ten weeks when the complete response of AVM was documented without any functional or aesthetic deficits. Long-term complete remission remained at the last follow-up visit 18 months after the treatment. Due to patient’s comorbidities, age and general health status no control arteriogram or MRI was preformed after the treatment to confirm complete clinical regression of AVM (**Figure 4**).

## Discussion

Several classification systems for vascular malformations have been proposed. The most comprehensive is the International Society for the Study of Vascular Anomalies (ISSVA) classification, which divides them into two main categories: tumors (true proliferative neoplasms) and malformations (morphogenetic defects) (20). Malformations are further subclassified into low-flow and high-flow lesions, depending on their hemodynamic characteristics (20–22).

AVMs, as high-flow vascular malformations, are the most challenging type of vascular malformations to manage, as they are associated with high recurrence rates and local destruction of surrounding tissues (23). A complete cure is challenging to obtain; consequently, treatment is often oriented toward controlling symptoms and preserving vital functions. The standard treatment options are embolization and/or surgical excision. AVMs can be embolized with different embolic agents either percutaneously or through a transarterial approach (1, 5, 10, 11, 24–26). Besides BLM, absolute ethanol is described as an effective embolic agent for treating AVMs of head and neck. Majority of reports state superiority of absolute ethanol when compared to BLM in treating low-flow vascular malformations (27). There are several studies reporting good treatment outcomes with absolute ethanol embolization administered either intralesionaly with direct puncture or transarterialy with microcatheterization (28, 29). Absolute ethanol alone has better overall response rate than BLM, but has notably more adverse events when the two are compared (30–32).

In a recently published study, BLM was effectively used to treat early-stage AVMs. BLM was injected intralesional rather than intravascularly since BLM cannot induce vascular disruption immediately, and its accumulation inside the vessels could be hampered in high-flow AVMs (33). In our study, we significantly bypassed BLM washout by combining ECT and superselective catheterization. Superselective catheterization has two essential advantages. BLM could be administered intra-arterially directly in the malformation vessels, and BLM is confined in the vessels during electroporation due to low-flow conditions achieved by balloon inflation.

We can hypothesize that BLM remained entrapped inside the AVM vessels because of the antivascular effects of electroporation. Vasoconstriction was clearly observed on arteriogram immediately after the procedure. In tumoral vessels stimulating the sympathetic nervous system and precapillary sphincters leads to an immediate vasoconstrictive response (“vascular lock effect”) lasting several hours. The vascular lock effect is followed by a delayed vascular disrupting effect (12, 34). Electroporation causes a transient increase in cell permeability through electropore formation in cell membranes, thus making endothelial cells more permeable to BLM. The result is the higher cytotoxicity of BLM, which causes apoptosis and necrosis of endothelial cells. Consequently, disruption of blood vessel continuity leads to a permanent reduction in blood flow (6, 8, 12, 13, 35, 36). This phenomena is regarded as reversible electroporation as opposed to irreversible electroporation (IRE) where higher amplitude and more electric pulses are used in order to achieve permanent cell membrane disruption and consequently induce cell death and necrosis (37–39). In this case ECT was preferred over IRE as there are many reports with good treatment outcomes when using this procedure on vascular malformations (10, 11). In addition, IRE is mostly used for treating deep-seated tumors and to our knowledge there have been no reports on treating vascular malformations with IRE (38, 39). Moreover as BLM is a well-established embolic agent for AVM treatment, ECT in combination with superselective catheterization was proposed as a potentially curative procedure.

The high sensitivity of endothelial cells to ECT was already demonstrated in preclinical and clinical studies (12, 40, 41). Interestingly, venules are more affected than arterioles, indicating that muscle cells in the arterial wall can tolerate more damage (42). These findings are in concordance with McMorrow et al., who demonstrated promising results in treating venous malformations with a reduced dose of BLM in combination with ECT (10, 13, 42). ECT is also used in treating vascular tumors such as Kaposi sarcoma with excellent results. In the treatment of Kaposi, objective and complete response rates are 100% and 92,8%, respectively (43). Such promising results support our concept of combining superselective catheterization and ECT as a possible efficient treatment of high-flow vascular malformations.

It is important to emphasize that we used 750 IU of BLM only once, in contrast to a study where up to 15 000 IU of intralesional BLM was used without electroporation every month for a total of 6 months in treating venous malformations (18). According to the Standard Operation Procedure (SOP) for ECT, the intratumoral dose of BLM should not exceed 250 IU BLM per 1 cm^3^ tumor volume. In our study the tumor volume was 5 cm^3^. Since the patient was old with several comorbidities and BLM was injected intra-arterially, we additionally reduced the recommended dose by 40% to avoid significant necrosis of the treated area and pulmonary fibrosis. This decision was based on our previous studies on elderly patients treated with ECT in combination with a reduced dose of intravenously administered BLM (8, 44, 45). Another significant issue is using superselective catheterization for intraarterial BLM administration and balloon occlusion to prevent BLM washout during electroporation. Indeed, such a procedure could only be performed in centers with an experienced interventional radiologist.

It should be noted that the patient has no functional deficits, such as oral incompetence. Even the final aesthetic result was excellent, despite the prolonged healing interval. Good functional and aesthetic outcomes are essential in the head and neck area. Furthermore, the good therapeutic outcome demonstrated in our study on small lower lip AVM confirms the efficacy of the combination of ECT and superselective catheterization in the treatment of this type of high-flow AVM. Compared with multiple treatments using BLM as an embolic agent without electroporation, our approach might allow a single treatment with a lower BLM dose to achieve a good clinical outcome. However, further studies on the application of our approach in AVM of different location and size are needed to confirm this approach compared with other embolization techniques. With this approach the possibility of adverse effects referring to BLM toxicity are significantly reduced. Nevertheless, it is important to emphasize the potential swelling after ECT. This could be relevant in the treatment of mucosal vascular malformations in the head and neck region, where oedema could pose serious health risks. We are aware that our procedure has several limitations, but on the other hand arises some relevant clinical questions such as what are optimal BLM concentrations in vascular malformations and still sufficient electric field to obtain complete response of vascular malformations with minimal intensive healing process. All these questions need to be addressed in further clinical studies.

## Conclusions

In conclusion, the technical advancements described in our study may improve the results of the high-flow vascular malformation treatment approach. Furthermore, with this technological advancement, cutaneous high-flow vascular malformations of the head and neck could be placed as an indication for ECT.

However, it is important to understand the healing process after ECT, since swelling and consequently feeding difficulties may occur when treating AVMs in the head and neck area. It is also important to note that this procedure can only be performed in centers where both ECT and experienced interventional radiologists are available.

## Data availability statement

The raw data supporting the conclusions of this article will be made available by the authors, without undue reservation.

## Ethics statement

The studies involving human participants were reviewed and approved by Slovenian National Medical Ethics Committee. The patients/participants provided their written informed consent to participate in this study.

## Author contributions

Conceptualization: AG and AK; Methodology: AG, DL, MC, CJ, and AK; Investigation: AK, AG, GS, and CJ; Data curation: AK, AG, and GS; Writing original draft: AK and AG; Writing, review and editing: AG and GS; Visualization: AK and AG; Supervision: AG and GS; Project administration: AK. All authors contributed to the article and approved the submitted version.

## Funding

This work was financially supported by the state budget by the Slovenian Research Agency, program no. P3-0003 and program no. P3-0307.

## Conflict of interest

The authors declare that the research was conducted in the absence of any commercial or financial relationships that could be construed as a potential conflict of interest.

## Publisher’s note

All claims expressed in this article are solely those of the authors and do not necessarily represent those of their affiliated organizations, or those of the publisher, the editors and the reviewers. Any product that may be evaluated in this article, or claim that may be made by its manufacturer, is not guaranteed or endorsed by the publisher.
